# P68 RNA Helicase (DDX5) Alters Activity of *Cis-* and *Trans*-Acting Factors of the Alternative Splicing of H-Ras

**DOI:** 10.1371/journal.pone.0002926

**Published:** 2008-08-13

**Authors:** Maria Camats, Sonia Guil, Mariette Kokolo, Montse Bach-Elias

**Affiliations:** Unidad de Splicing, Instituto de Investigaciones Biomédicas de Barcelona-Consejo Superior de Investigaciones Científicas, Barcelona, Spain; Centre de Regulació Genòmica, Spain

## Abstract

**Background:**

H-Ras pre-mRNA undergoes an alternative splicing process to render two proteins, namely p21 H-Ras and p19 H-Ras, due to either the exclusion or inclusion of the alternative intron D exon (IDX), respectively. p68 RNA helicase (p68) is known to reduce IDX inclusion.

**Principal Findings:**

Here we show that p68 unwinds the stem-loop IDX-rasISS1 structure and prevents binding of hnRNP H to IDX-rasISS1. We also found that p68 alters the dynamic localization of SC35, a splicing factor that promotes IDX inclusion. The knockdown of hnRNP A1, FUS/TLS and hnRNP H resulted in upregulation of the expression of the gene encoding the SC35-binding protein, SFRS2IP. Finally, FUS/TLS was observed to upregulate p19 expression and to stimulate IDX inclusion, and *in vivo* RNAi-mediated depletion of hnRNP H decreased p19 H-Ras abundance.

**Significance:**

Taken together, p68 is shown to be an essential player in the regulation of H-Ras expression as well as in a vital transduction signal pathway tied to cell proliferation and many cancer processes.

## Introduction

H-, K- and N-Ras proteins play a significant role in controlling cell growth, a central component of mitogenic signal transduction pathways, reviewed in [1], [2]. So far, H*-ras* knockout (KO) mice were found to be viable, indicating that H-*ras* is not required for embryogenesis [3]. However, H-*ras* mutations were shown to be an important hallmark in Costello syndrome, an extremely rare disorder that affects multiple organ systems [4], [5]. H-Ras pre-mRNA undergoes an alternative splicing process to render two proteins, namely p21 H-Ras and p19 H-Ras, a process driven by the exclusion or inclusion of the alternative intron D exon (IDX), respectively [6], [7], [8]. Due to the presence of an in-frame stop codon within IDX, p19 mRNA is translated into a shorter protein, named p19 H-RasIDX or p19 [7]. P19 is ubiquitous and conserved in all mammalian species, where it localizes in the nucleus and cytosol. Interestingly, unlike p21, p19 does not bind to GTP, thereby indicating that these two Ras proteins have distinct and complementary roles. P19 is also known to bind to RACK1 [7], a scaffolding protein that brings together different factors, enabling them to act in a common pathway, e.g. mitogen-activated protein kinase pathways [9], [10]. Moreover, interaction between p19 and p73β was shown to decrease the MDM2-mediated transcriptional repression of p73β [11]. Finally, recent studies have indicated that p19 regulates G1/S cell cycle progression (Camats et al. unpublished).

**Figure 1:**
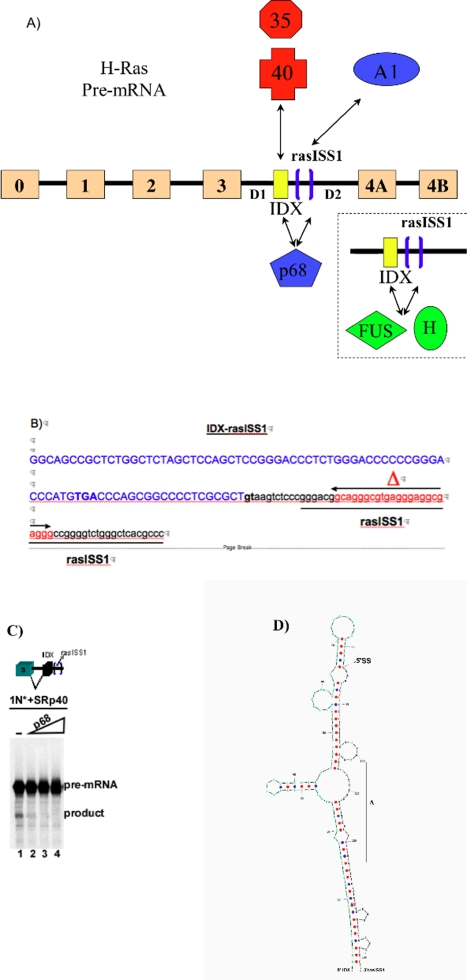
Recently proposed model for the regulation of alternative splicing for H-Ras pre-mRNA by Guil et al. [8]. A) RasISS1 (blue brackets) is the silencer downstream of the alternative intron D exon (IDX), showed as an yellow exon. Arrow-headed lines indicate protein-RNA interactions. Red factors activate IDX inclusion (SC35 and SRp40); blue factors inhibit IDX inclusion. Concerning to the green factors, only direct/indirect binding to both IDX and rasISS1 sequences has been reported. P68 RNA helicase also showed binding to both IDX and rasISS1 sequences. For clarity, FUS/TLS- and hnRNP H-RNA bindings are separately showed below on the right. B) Detail of the IDX and rasISS1 sequences. “Δ” indicates the region deleted in ΔrasISS1. C) *In vitro* splicing reaction of 1N pre-mRNA (containing exon3-D1-IDX-rasISS1) activated with 400 ng of recombinant SRp40 (lanes 1–4). For lanes 2, 3 and 4, 100, 200 and 400 ng of recombinant p68 was added, respectively. The “*” indicates that ^32^P-labeled RNA was used in the assay. D) Proposed secondary structure of the IDX-rasIS1 sequence as previously published [8]. S. Guil et al. also demonstrated that this structure is conserved in hamster, mouse, rat and humans, and that some silent mutations are compensated by a silent one on the opposite strand across the four species [8]. The “Δ” indicates the region deleted in the ΔrasISS1.

Previously, we have characterized the cis-acting sequences and trans-splicing factors involved in the regulation of IDX inclusion to enable its study both *in vitro* and *in vivo* [8]. As previously shown, an intronic silencer sequence (named rasISS1) located downstream of IDX (see Figure 1A and 1B) negatively regulates upstream intron splicing [8]. This inhibitory effect was observed to be partially mediated by the binding of hnRNP A1, an inhibitor of IDX inclusion. The SR proteins SC35 and SRp40 are known to antagonize the negative action of hnRNP A1 stimulating IDX inclusion [8]. Additionally, hnRNP H, FUS/TLS and the p68 RNA helicase (p68) are also associated with both IDX and rasISS1 RNA, suggesting a role for these proteins as bridging factors between IDX and rasISS1. A stem-loop structure within the IDX-rasISS1 sequence (see Figure 1B and 1C) was suggested to have a regulatory role in this IDX inclusion/exclusion, a notion which is reinforced by the observation that the knockdown of p68 increases IDX inclusion in endogenous p19 mRNA. In this work, we have further characterized the role of p68 in p19 regulation. Using winding/unwinding assays, we analyzed how p68 affects IDX-rasISS1 stem-loop structure and the binding of hnRNP H and FUS/TLS to this IDX-rasISS1 region. RNAi-mediated depletion experiments showed how hnRNP A1, FUS/TLS, hnRNP H and p68 alter the activity and dynamic localization of SC35 and H-ras alternative splicing. Here we present an updated and extended version of the alternative splicing model for H-Ras regulation with special emphasis on the role of p68 as regards its role in this vital transduction signal pathway tied to cell proliferation and many cancer processes.

## Results and Discussion

Figure 1A depicts a recently proposed model for the regulation of alternative splicing of pre-mRNA H-Ras in which hnRNP A1 and p68 act as inhibitors and SC35 and SRp40 as stimulators of IDX inclusion [8]. In this model, there are two distinguishing features driving IDX inclusion: (1) the silencer sequence (rasISS1) downstream of IDX that contains a hnRNP A1-binding site; and (2) the IDX itself also harbors putative sequences that can bind SC35 and SRp40. Previously, studies using RNA affinity columns containing either IDX, rasISS1 or the mutant ΔrasISS1 (see Figure 1B) in presence of nuclear extract showed that p68, hnRNP H and FUS bound to IDX and rasISS1, but not to ΔrasISS1 [8]. Previous *in vivo* studies found that RNAi-mediated depletion of p68 increased the level of endogenous p19 mature mRNA, thereby revealing a role for p68 RNA helicase as an inhibitor of IDX inclusion [8]. Here we show that the addition of recombinant p68 to *in vitro* splicing reactions (Figure 1C) inhibits splicing of intron D1, (lanes 2, 3 and 4) and reverts the activation of SRp40 (compare lanes 1 to 2, 3 and 4) confirming previous results obtained *in vivo* [8].

**Figure 2:**
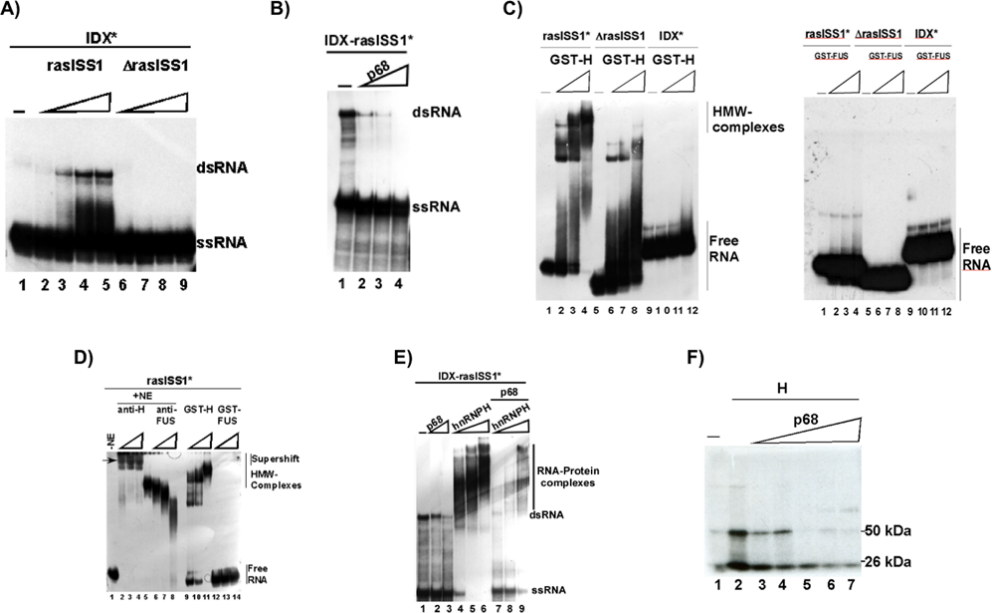
P68 RNA helicase unwinds IDX-rasISS1 *in vitro*. A) Winding activity assay between IDX and rasISS1 RNAs. The “*” indicates that ^32^P-labeled RNA was used. The other RNAs were added unlabeled (cold). All lanes contain 18 ng IDX. Lane 1: no additional RNA; lanes 2, 3, 4 and 5: 1, 10, 50 and 100 ng cold rasISS1 RNA, respectively; lanes 6, 7, 8 and 9: 1, 10, 50 and 100 ng cold ΔrasISS1, respectively. dsRNA: double-stranded RNA; ssRNA: single-stranded RNA. Samples were run on a 8% (w/v) native polyacrylamide gel. B) Winding activity assay of the IDX-rasISS1 RNA (1 pmol) in the absence (lane 1) or presence of increasing amounts of p68 (100, 200 and 400 ng in lanes 2, 3 and 4, respectively). P68 RNA helicase was added after winding assays followed by 1 h at 37°C. Samples were run on a 8% (w/v) native polyacrylamide gel. C) On the left, binding-shift assay of labeled rasISS1, ΔrasISS1 and IDX RNAs (1 pmol each) with increasing amounts of recombinant GST-fused hnRNP H (GST-H). Lanes 1, 5 and 9 did not contain GST-H; lanes 2–4, 6–8 and 10–12 contained 100, 200 and 400 ng, respectively, of recombinant GST-H. HMW (high molecular weight). On the right, similar experiment with GST-FUS is showed. D) Binding-shift assay of labeled rasISS1 RNA in nuclear extract (lanes 2–8) or without extract (in Roeder D buffer, lanes 9–14). Lane 1 did not contain nuclear extract. To obtain supershift complexes, 0.5, 1 and 2 µl anti-hnRNP H serum (lanes 2, 3 and 4, respectively) and 0.5, 1 and 2 µl anti-FUS (lanes 6, 7 and 8, respectively) were added to the reaction. Lane 5 contained nuclear extract only. Lanes 9–10, 11–12 and 13–14 contain 100, 200 and 400 ng of recombinant GST-H and GST-FUS, respectively. Samples were run on a 6% (w/v) native polyacrylamide native gel. HMW (high molecular weight). E) Pre-winded ^32^P-IDX-rasISS1 RNA was incubated with 0.6 and 1.2 µg recombinant p68 (lanes 2 and 3, respectively), with 160, 320 and 640 ng recombinant hnRNP H (lanes 4, 5 and 6, respectively). Lanes 7, 8 and 9 contained 160, 320 and 640 ng recombinant hnRNP H, respectively plus 1.2 µg of recombinant p68. Samples were run on a 6% (w/v) native polyacrylamide gel. F) Crosslinking assay between hnRNP H (640 ng) and ^32^P-IDX-rasISS1 RNA (1 pmol) (lanes 2–7). Lane 1 did not contain hnRNP H. Lanes 3–7 contained 100, 200, 400, 800 and 1200 ng p68, respectively. Samples were run on a 10% SDS-polyacrylamide protein gel and autoradiographed.

RNA folding predictions suggested a stem-loop secondary structure comprising IDX and rasISS1 sequences, see [8] and figure 1D, that is conserved between hamster, mouse, rat and human [8]. Interestingly, the addition of the stem-loop RNA sequence to *in vitro* splicing reactions enhanced IDX inclusion [8], suggesting that the reaction can be activated by titrating out inhibitory factors that recognize this structure. To gain additional insights into the function of this putative stem-loop structure, we tested whether both individual IDX and rasISS1 sequences wind together *in vitro*. Figure 2A clearly shows that double-stranded (dsRNA) structures were obtained for IDX and rasISS1 in RNA native gels (see lanes 2–5), but, very interestingly, not for the ΔrasISS1 mutant (lanes 6–9), thereby indicating that the“Δ” sequence is essential for the formation of this dsRNA structure. We next studied whether the IDX-rasISS1-linked sequence winds and forms a dsRNA *in vitro* and found that the IDX-rasISS1 sequence wound in a dsRNA structure (Figure 2B, lane 1) that was rapidly reverted to ssRNA in the presence of p68 (lanes 2, 3 and 4). This finding demonstrates that IDX-rasISS1 winds in a dsRNA structure that is recognized and can be unwound by p68, pointing to a role for this secondary structure in the efficient inclusion of the alternative exon IDX.

The intrinsic nature of the binding of hnRNP H and FUS/TLS to IDX and rassISS1 sequences was further studied using binding-shift assays. GST-fused hnRNP H (GST-H) showed a binding-shift in native gels in the presence of the rasISS1 sequence, reflecting the formation of high molecular weight complexes (Figure 2C, left lanes 2, 3 and 4), but not in the presence of ΔrasISS1 (Figure 2C, lanes 6, 7 and 8). Since hnRNP H showed no binding-shift with the IDX sequence (Figure 2C, lanes 10–12) we conclude that its previously reported association to IDX RNA-affinity [8] columns is due to an indirect binding or it is a weak interaction that does not survive gel resolution in binding shift assays. Our studies further demonstrate that rasISS1 forms a protein-RNA complex in nuclear extract (Figure 2D, lane 5) that is stabilized and supershifted with anti-hnRNP H antibodies (lanes 2, 3 and 4), thereby indicating that hnRNP H is present in this rasISS1 complex and that it is highly accessible to the antibody. HnRNP H also showed a binding-shift with the stem-loop IDX-rasISS1 (Figure 2E, lanes 4–6) that was weakened by the unwinding activity of p68 (see lanes 7–9). Moreover, crosslinking assays between labeled hnRNP H and IDX-rasISS1, in the absence or presence of p68 under splicing conditions *in vitro,* further confirmed that hnRNP H and IDX-rasISS1 directly bind (see band at around 50 kDa: Figure 2F, lane 2) and that this binding is inhibited in the presence of p68 (Figure 2F, lanes 3–7). Collectively, data from the binding-shift and cross-linking assays indicate that (1) hnRNP H directly binds to rasISS1 and that the “Δ” sequence is required for this binding (Figure 2C, lanes 1–8); (2) hnRNP H does not directly bind to the IDX sequence and therefore (Figure 2C), as it was seen to bind in previous IDX-RNA-affinity column studies [8], we propose that hnRNP H indirectly binds to IDX via at least one other IDX-bound protein in the complex. A second explanation is that this binding is weak and does not survive gel resolution in binding shift assays; (3) hnRNP H was seen to directly bind to the stem-loop IDX-rasISS1 that involves a dsRNA stem-loop structure (Figure 2E, lanes 4–9); and (4) p68 regulates hnRNP H-stem-loop binding since p68 reduces this interaction (Figure 2E and 2F).

**Figure 3:**
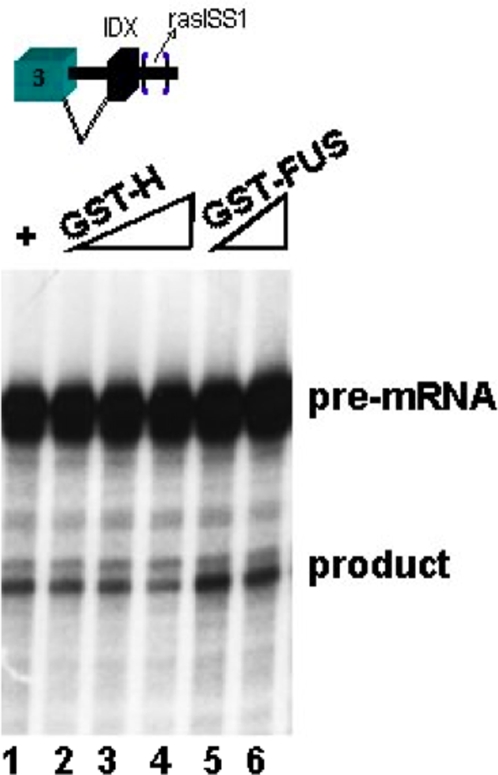
FUS stimulates IDX inclusion. Lane 1: *In vitro* splicing reaction of ^32^P-1 N pre-mRNA (containing exon3-D1-IDX-rasISS1); lanes 2, 3 and 4: 65, 130 and 260 ng recombinant GST-H,; lanes 5 and 6: 90 and 180 ng recombinant GST-FUS. Samples were run on a 10% urea denaturing polyacrylamide gel.

Although FUS/TLS was found to bind to IDX and to rasISS1 RNA-affinity columns, this protein showed no binding-shift to any IDX or rasISS1 sequence (Figure 2C, right). These results indicate that FUS/TLS should therefore indirectly bind to IDX and rasISS1 through other IDX-bound proteins. Again, a second explanation is that the binding may be weak and does not survive gel resolution in gel shift assays. Furthermore, *in vitro* splicing assays showed that GST-FUS stimulates intron D1 splicing (Figure 3, lanes 5 and 6), whereas GST-H did not (lanes 2, 3 and 4).

**Figure 4:**
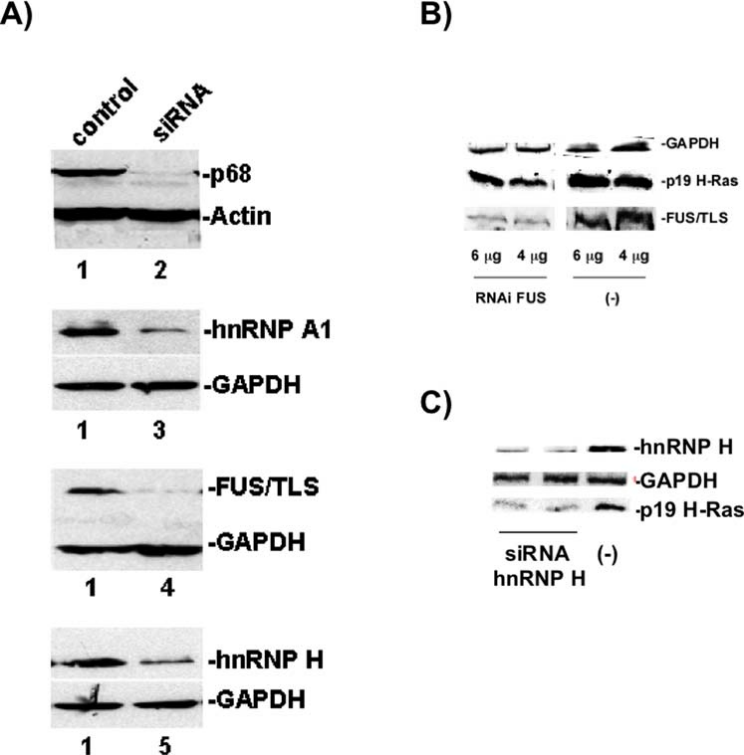
RNAi-mediated depletion of FUS and hnRNP H downregulates p19 H-Ras expression. A) Western blot on HeLa extracts from cells specifically depleted by means of RNAi-mediated depletion of p68 (lane 2), hnRNP A1 (lane 3), FUS/TLS (lane 4) and hnRNP H (lane 5). Lanes 1: HeLa cells transfected with empty pSuper vector (negative control). Antibodies against actine or GAPDH were used as loading controls. B) Western blot on extracts similar as in (A), lane 4, with RNAi-mediated depletion of FUS/TLS (lanes RNAi FUS) as compared to cells transfected with empty vector, lanes (-). Cells were transfected with 6 µg or 4 µg of the pSuper plasmids. Samples were run on 12.5% SDS denaturing polyacrylamide gels. Blots were incubated with anti-p19 and anti-FUS antibodies. Anti-GAPDH was used in all lanes as internal loading control and also as a fixed value of 1 to which all calculations for each lane were standardized. Blots subjected to chemiluminescence (peroxidase-luminol) and then quantified with the imaging software Multi Gauge V3.0 (Fujifilm) using LAS 3000 apparatus (Fujifilm). The RNAi-mediated depletion of FUS/TLS induced a drop on the protein abundance of 52–64% on FUS and 27–35% on p19. C) Western blot on extracts with siRNA-mediated depletion of hnRNP H with a siRNA to hnRNP H (Santa Cruz Biotechnology, sc-35579). Lane (-) contains the siRNA-B control (Santa Cruz Biotechnology, sc-44230). The siRNA depletion of hnRNP H induced a drop on the protein abundance of 50% on both hnRNP H and p19 proteins.

To better define the roles of hnRNP A1, hnRNP H, FUS/TLS and p68 in regard to p19 as well as other genes, we analyzed the effect of depleting these four splicing factors by means of RNAi-mediated depletion assays to determine how (1) endogenous p19 abundance is affected and (2) the expression of other genes is regulated. The RNAi-mediated depletion of the four splicing factors were obtained with specific shRNA oligonucletides (Figure 4, lanes 2–5) using HeLa cell extracts. Previously, we have shown that RNAi-mediated depletion of p68 increases p19 mRNA levels [8]. In this study, we found that RNAi-mediated depletions of FUS/TLS or hnRNP H decreased the p19 protein level as seen by Western blot quantification (see Figure 4B and C, respectively); a finding that corroborates with our observation that the efficient splicing of intron D1 is proportional to the FUS/TLS level (Figure 3). No change in p19 mRNA abundance was seen during the RNAi-mediated depletion of hnRNP A1 albeit different interfering sequences were used (result not shown). As hnRNP A1 was previously reported to downregulate p19 [8], we suggest that we did not observe this effect for two main reasons: (1) the level of hnRNP A1 is high and the remaining amount of hnRNP A1 is enough to maintain the level of p19 mRNA; and (2) other hnRNP proteins similar to A1, (e.g. A2/B1 and A3 have 57% and 85% homology, respectively), can also participate in and therefore compensate for lower levels of A1 in regards to regulation of alternative splicing of H-Ras pre-mRNA.

The RNA derived from the four RNAi-mediated depletion assays of the splicing factors in HeLa cells were individually incubated in microarrays containing 19,000 human ESTs. From the results obtained, we sorted the genes exhibiting modified expression into five groups showed in Table S1. Group I contains genes whose expression varies with both the RNAi-mediated depletion of hnRNP A1 and p68, but not with the RNAi-mediated depletion of FUS/TLS and hnRNP H. Groups II to V have genes whose expression only varies with the RNAi-mediated depletion of one of the four splicing factors, but not with the RNAi-mediated depletion of the other three. Interestingly, only the *ZNF462* gene was found to increase its expression in the presence of RNAi-mediated depletion of hnRNP A1 and p68. ZNF462 is a zinc finger protein that has been implicated in agenesis of the corpus callosum [12], a common brain anomaly. We suggest that ZNF462 and p19 H-Ras could be coordinately regulated by hnRNP A1 and p68. Microarrays analyzing the RNAi-mediated depletion of hnRNP A1 showed an increase and decrease of *PDCD4* and *BAG4 mRNA* levels, respectively; the proteins of which are thought to have anti-apoptotic effects [13], [14]. For the RNAi-mediated depletion of FUS/TLS, the microarray data showed a decrease in the tyrosine protein kinase *EGFR* mRNA level; *EGFR* gene mutations have been observed in lung cancers [15]. Moreover, RNAi-mediated depletion of hnRNP H also produced a significant decrease in the *ARAF, CABLES and PCBP4* mRNA levels. All three proteins are known to be involved in cell proliferation [16], [17], [18]. Interestingly, the knockdown of p68 downregulated *TOB2* that is known to be an endogenous activator of pro-proliferative pathways [19].

Since the SR proteins SC35 and SRp40 are known to enhance p19 H-Ras alternative splicing [8], we also analyzed whether the RNAi-mediated depletion of hnRNP A1, FUS/TLS, hnRNP H and p68 affect SR proteins mRNA levels. Selected results of these studies are provided in Table 1. Note that other SR proteins that showed no variation were not included in the table. The mRNA levels of SC35 and SRp40 showed no variation with any of the RNAi-mediated depletions here studied; however, interestingly, SRp20 and SFRS14 mRNAs were downregulated with RNAi-mediated depletion of p68. Although SC35 was not regulated by any of these RNAi-mediated depletions, a SR protein that specifically binds to SC35, namely SFRS2IP, was upregulated by RNAi-mediated depletion of hnRNP A1, and to a lesser extent by the RNAi-mediated depletions of FUS/TLS and hnRNP H. These findings suggest that these three proteins may regulate a feedback loop involving SC35 via SFRS2IP.

**Table 1:** *Log_2_* is the log_2_ value of the fold change measuring the effect of the RNAi on the ESTs expresión as compared with the negative control performed with the empty pSuper vector.

SR proteins expression affected by RNAi of hnRNP A1, FUS/TLS, hnRNP H and p68 RNA helicase				
A1	FUS	H	P68	SR protein symbol
*Log_2_*				
0.00	−0.14	0.00	−0.82	SFRS3 (SRp20)
−0.40	−0.07	−0.32	0.29	SFRS2 (SC35)
−0.36	0.00	−0.04	−0.04	SFRS5 (SRp40)
0.25	0.08	−0.38	−0.88	SFRS14
1.44	0.85	0.91	0.51	SFRS2IP

*Log_2_* higher than 1 were taken as significant values.

**Figure 5:**
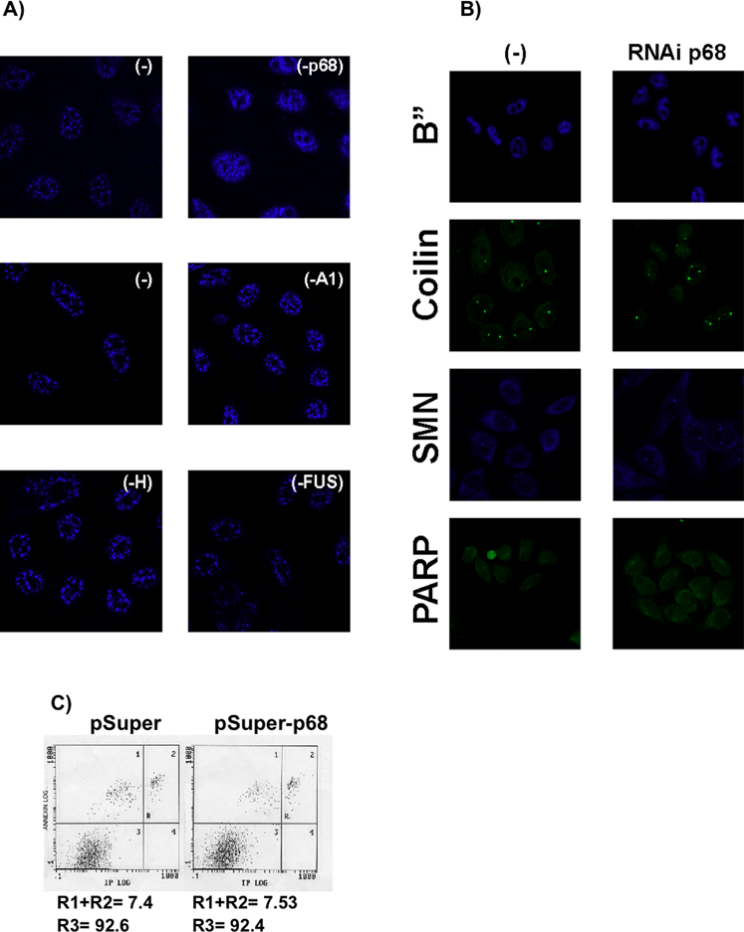
Downregulation of p68 RNA helicase alters the dynamic localization of the SC35 splicing factor. A) Indirect immunofluorescence detection of HeLa cells containing the pSuper-RNAi-p68 RNA helicase vector (-p68); pSuper-RNAi-A1 vector (-A1); pSuper-RNAi-H vector (-H); pSuper-RNAi-FUS vector (-FUS). As negative controls, cells were transfected with empty pSuper (-). Anti-SC35 primary antibody diluted 1/600 (mouse) and secondary anti-mouse Alexa Fluor® 633 F(ab')_2_-labeled antibody . B) Indirect immunofluorescence detection of HeLa cells containing the pSuper-RNAi-p68 RNA helicase vector (-p68) or pSuper empty vector (-) incubated with anti-B” (1/20, mouse), anti-Coilin (1/100, rabbit), anti-SMN (1/1000, mouse) and FITC-labelled anti-PARP (1/100, mouse). Primary mouse and rabbit antibodies were incubated with secondary anti-mouse Alexa Fluor® 633 or 488, respectively, F(ab')_2_-labeled antibody. C) Flow cytometric experiments estimating apoptosis levels in HeLa cells transfected either with empty pSuper or pSuper-RNAi-p68 RNA helicase vectors, and stained with annexin V-FITC (Annexin LOG) and propidium iodide (IP LOG). R3 represents % of viable cells and R1+R2 % of dead plus apoptotic cells.

Using RNAi-mediated depletion assays, we next analyzed how the RNAi-mediated depletions of p68, hnRNP A1, hnRNP H and FUS/TLS affect SC35 localization inside the cell. We found that only RNAi-mediated depletion of p68 alters SC35 localization (Figure 5A, panel -p68) by increasing SC35 levels outside of the interchromatin granule cluster (IGC) in a diffuse nuclear pattern. This suggests that RNAi-mediated depletion of p68 can induce SC35 to be localized similar to that seen in actively transcribing cells where both splicing and transcription factors display a highly dynamic localization within the nucleus and may be recruited from speckles to the position of active transcription [20]. Moreover, despite not affecting SC35 expression, the downregulation of p68 may increase the level of SC35 protein available for the splicing machinery, such as for p19 H-Ras alternative splicing.

Whether p68 modifies the dynamic localization of other speckles was also studied in RNAi-mediated depletion assays. RNAi-mediated depletion of p68 was observed not to affect the localization of B” (U2snRNP), Coilin and SMN speckles; however, when p68 was knocked down, SMN bodies were more susceptive to anti-SMN antibodies (Figure 5B, compare SMN panels). Finally, RNAi-mediated depletion of p68 did not stimulate cell apoptosis (Figure 5B PARP panels and 5C), nor did it activate the dsRNA-activated protein kinase PKR, since eIF2-α was not hyperphosphorylated (not shown).

Collectively, we have found that p68 regulates p19 H-Ras splicing by (1) affecting the dsRNA structure of the stem-loop IDX-rasISS1 (Figure 2B), (2) disrupting binding of hnRNP H to the stem-loop IDX-rasISS1 (Figure 2E) and (3) may increase the level of SC35 protein available for the splicing machinery, such as for p19 H-Ras alternative splicing (Figure 5A). Moreover, (4) knockdown of either hnRNP A1, FUS/TLS or hnRNP H (Table 1) led to upregulation of SFRS2IP, a SC35-binding protein. Currently, studies are underway to decipher whether SFRS2IP exerts an effect on IDX inclusion. Finally, (5) although FUS/TLS showed no direct binding to the stem-loop IDX-rasISS1 (Figure 2 C, right), assays have indicated that FUS stimulated IDX inclusion (Figure 3 and Figure 4B). (6) Now, we know that hnRNP H binds to the stem-loop IDX ras-ISS1 (Figure 2), but so far our *in vitro* splicing studies have not thrown light on its action as regards IDX inclusion. However, as the p68 (an inhibitor of IDX inclusion) weakened hnRNP H binding to the stem-loop (Figure 2), it is likely that hnRNP H is necessary to stimulate IDX inclusion. This latter reasoning agrees with the *in vivo* downregulation of p19 by hnRNP H knock-down (Figure 4 C).

There are to date only a few studies reporting on the synchronized action of all the splicing factors studied here. Interestingly, RNA helicase A was also found to associate with hnRNP proteins including hnRNP H [21]. Additionally, RNAi-mediated depletion studies revealed that by suppressing the expression of RNA helicase A, the nuclear distribution of hnRNP C was altered [21]. Van Herreweghe *et al.* recently showed that a fraction of HeLa 7SK snRNA specifically interacts with RNA helicase A and heterogeneous nuclear ribonucleoprotein A1 [22]. Moreover, p68 was found to shuttle in and out of SC35 domains, forming fibers and granules in a cell-cycle dependent manner [23]. Functional analysis demonstrated that SC35 and TASR, a FUS-associated protein with SR repeats, have antagonistic effects on adenovirus E1A pre-mRNA splicing and abrogate the influence of FUS on this splicing. Interestingly, protein-protein assays have revealed that FUS is also associated with helicases [24]. The hnRNP A1 and SR proteins have also been reported to have antagonistic functions in splicing processes [8], [25], [26], [27], [28]. In addition, a recent NMR study showed the competition between proteins SC35, SRp40 and heterogeneous nuclear ribonucleoprotein A1 at the HIV-1 Tat exon 2 splicing site [26].

All the factors implicated so far in the regulation of the alternative splicing of H-Ras seem to also be involved in regulating many other splicing processes and to act in concert. An observation that serves to emphasize that the alternative splicing model studied here is highly helpful in advancing our understanding of the interactions of all these splicing factors. This enhanced understanding will better enable us to define *cis*-element sequences and *trans*-acting factors as well as to determine their interactions in generating future models for other exon splicing processes. The number of *cis*-element sequences and *trans*-acting factors implicated in the alternative splicing of H-Ras pre-mRNA, as well as RNA secondary structures, indicates how complex and fine-tuned the regulation of this gene is. The pivotal role of p68 in the regulation of such an important signal transduction pathway further underlines the need to better understand winding/unwinding activities in cancer processes.

## Materials and Methods

### Antibodies

The following antibodies were generous gifts: anti-p68 RNA helicase PAb204 (Dr. F. Fuller-Pace); anti-hnRNP A1 4B10 (Dr. G. Dreyfuss); anti-hnRNP H AN113 (Dr. D. Black); anti-FUS/TLS 474 (Dr. F. Moreau-Gachelin); anti-SMN 2B1 (Dr. G. Dreyfuss); and anti-p19 H-Ras [7]. Anti-SC35 (BD Pharmingen), anti-B” (Euro-Diagnostica), anti-Coilin C1862 (Sigma) and anti-PARP (BD Pharmingen) were purchased from commercial suppliers.

### Plasmids constructions, *in vitro* splicing assays, transfection assays and RNA analysis, gel shift assays, and SR protein complementation and western blots

Details of all constructs and approaches were previously reported [7], [8].

### Native RNA gels and winding/unwinding assays

^32^P-labeled (1 pmol; 20,000 cpms) RNAs (either IDX plus rasISS1 or IDX-rasiSS1) were placed to wind in Roeder D buffer containing 1 mM ATP and 1.5 mM MgCl_2_ for 5 min at 90°C followed by 1 h at 37°C. P68 RNA helicase and/or hnRNP H were added after winding assays followed by 1 h at 37°C. The reactions were stopped by the addition of 50% glycerol, 2% SDS, 20 mM EDTA and 0.025% bromophenol blue and xylene cyanol (25% of the total reaction volume). Samples were analyzed on 8% (w/v) native acrylamide gels in 1x TBE buffer.

### RNAi

The deoxyoligonucleotides used to construct pSuper are as follows:

pSUPER-FUS605for, gatccccGATCAATCCTCCATGAGTAttcaagagaTACTCATGGAGGATTGATCtttttggaaa;

pSUPER-FUS605rev, agcttttccaaaaaGATCAATCCTCCATGAGTAtctcttgaaTACTCATGGAGGATTGATCggg;

pSUPER-H323for, gatccccGTATTCAAGTCAAACAACGttcaagagaCGTTGTTTGACTTGAATACtttttggaaa;

pSUPER-H323rev, agcttttccaaaaaGTATTCAAGTCAAACAACGtctcttgaaCGTTGTTTGACTTGAATACggg;

pSUPER-A1382for, gatccccGATTCTCAAAGACCAGGTGttcaagagaCACCTGGTCTTTGAGAATCtttttggaaa;

pSUPER-A1382rev, agcttttccaaaaaGATTCTCAAAGACCAGGTGtctcttgaaCACCTGGTCTTTGAGAATCggg.

pSuper-p68 RNA helicase vector and RNAi-mediated depletion assays were performed as previously described [8].

### Recombinant proteins

Recombinant plasmids were generous gifts from the following researchers: p68 was obtained from the pET30a vector (Dr. Z-R. Liu); GST-H from pGEX-H (Dr. J. Wilusz); GST-FUS from pGEX-FUS (Dr. F. Moreau-Gachelin); and hnRNP H from pET 15-B (Dr. D. Black). Recombinant proteins were obtained and purified as described by the manufacturers of the pGEX and pET vectors from AmershamBiosciences and Clontech, respectively.

### Direct and indirect immunofluorescence

These assays were performed as detailed elsewhere [7].

### Apoptosis assays

HeLa cells were transfected either with the pSuper-p68 RNAi or empty pSuper plasmids. Apoptotic cells were detected by flow cytometry using the Annexin V-FITC Apoptosis Detection Kit (Sigma) according to the manufacturer's instructions. Flow cytometric experiments were carried out using an Epics XL flow cytometer (Coulter Corporation, Miami, Florida, USA). Excitation of the samples was achieved by using an air-cooled argon-ion laser set at 488 nm and 15 mW power with other parameters at standard configuration. Forward scatter (FSC), side scatter (SSC), green (525 nm) fluorescence for Annexin-FITC-conjugated antibody and red (675 nm) fluorescence for PI were used. Green fluorescence was measured with a 550 nm dichroic-long filter and 525 nm bandpass filter. Red fluorescence was collected with a 645 nm dichroic-long filter and a 675 nm bandpass filter. Optical alignment was based on an optimized signal from 10 nm fluorescent beads (Immunocheck, Epics Division).

### ESTs microarrays incubation and bioinformatics analysis

cDNA samples from HeLa cells transfected with either pSuper-68 RNAi vector, pSuper-A1 RNAi vector, pSuper-H RNAi vector or pSuper-FUS RNAi vector were compared against control samples transfected with empty vector pSuper. Two-color hybridizations were performed in triplicate. A total of 12 human 19,008 different ESTs (ss-H19k4.1) from Microarray Centre (Toronto) were assayed. Briefly, 500 ng of total RNA from each sample were amplified by Oligo-dT-T7 reverse transcription and labeled by *in vitro* transcription with T7 RNA polymerase in the presence of Cy5-CTP or Cy3 CTP using the Low Input RNA labeling kit (Agilent) and cleaned using RNAeasy columns (Qiagen). After fragmentation, 750 ng of labeled cRNA from each of two samples were co-hybridized *in situ* in hybridization buffer (Agilent) for 18 h at 60°C and washed for 10 min in 6X SSC+0.005% Triton X-102 at room temperature and 5 min in 0.1X SSC+0.005% Triton X-102 at 4°C, followed by drying by centrifugation for 5 min at 1000 rpm. Images were generated on an Agilent confocal microarray scanner at 10 µm resolution and quantified using GenePix 6.0. Spots with signal intensities twice above the local background that were also not saturated or flagged by GenePix were considered as reliable and were given a weight of 1 for normalization purposes, whereas the rest were given weights of 0.01. Extracted intensities were subtracted from the local background and the log_2_ ratios were normalized in an intensity-dependent fashion by the global lowest method with a span parameter of 0.3. Normalized log_2_ ratios were scaled between arrays to allow better comparison of data. Raw data were processed using MMARGE (Lozano et al, unpublished), a web implementation (http://nin.crg.es/microarray.html) of Limma [29] and a microarray analysis library developed within the Bioconductor project in the R statistical environment [30]. For determining significant hits, the empirical Bayes statistic B was used. To compare the empty vector and RNAi vectors, a contrast test was applied taking the normalized log_2_ ratios of the three microarray hybridizations of each type as replicates against the same common control reference using BANAL, a web-based interface also running on Limma.

### Crosslinking assays

^32^P-labeled (1 pmol, 20,000 cpms) IDX-rasISS1 RNA was allowed to bind to 640 ng recombinant hnRNP H in binding buffer (1 mM ATP, 1.7 mM MgCl2 and 0.1 µM heparin in 1×Roeder D buffer). Binding reactions were also performed in the presence of increasing concentrations of recombinant p68 (100–1200 ng protein). All tubes were incubated for 30 min at 30°C, UV-crosslinked with a Stratalinker 1800 (Stratagene) at 1.2 J for 30 s and then treated with RNAse T1 for 1 h at 37°C. Proteins were analyzed on 8% SDS-PAGE gels as previously described [8] and then autoradiographed.

## Supporting Information

Table S1 Table S1 is loaded as online supporting information. The accession number for the microarray data is GSE12058 in http://www.ncbi.nlm.nih.gov/geo.
(0.14 MB DOC)
