# Supplementary material for: P68 RNA Helicase (DDX5) Alters Activity of Cis- and Trans-Acting Factors of the Alternative Splicing of H-Ras
Source: PLoS One. 2008 Aug 13;3(8):e2926. doi: 10.1371/journal.pone.0002926 (PMC2491553; doi:10.1371/journal.pone.0002926)
Supplement: Table S1 — is loaded as online supporting information. The accession number for the microarray data is GSE12058 in http://www.ncbi.nlm.nih.gov/geo. (0.14 MB DOC) [file pone.0002926.s001.doc]

TABLE S1

| Group I  Genes that vary with both, RNAi of hnRNP A1 and RNAi of p68 RNA helicase | | | | | |
| --- | --- | --- | --- | --- | --- |
| A1 | FUS | H | P68 | Accession Number | Gene  Name |
| *Log_2_* | | | |
| 1.06 | 0.18 | 0.14 | 1.15 | NT_008470 | *ZNF462* |

| Group II  Genes that only vary with RNAi of hnRNP A1 and not with RNAi of FUS/H/p68 | | | | | | | |
| --- | --- | --- | --- | --- | --- | --- | --- |
| A1 | FUS | | | H | P68 | Accession Number | Gene  Name |
| *Log_2_* | | | | | |
| 2.33 | | -1.51 | 0.75 | | 0.66 | NW_925006 | *C11orf69* |
| 1.73 | | -0.14 | 0.37 | | 0.06 | NM_000918 | *P4HB* |
| 1.61 | | 0.08 | 0.44 | | 0.26 | NW_925517 | *GPC6* |
| 1.59 | | 0.78 | 0.48 | | 0.60 | NW_921618 | *ABI2* |
| 1.51 | | 0.74 | 0.72 | | -0.58 | NT_030059 | *PDCD4* |
| 1.51 | | 0.63 | 0.75 | | 0.49 | NW_921351 | *RAB42* |
| -1.07 | | 0.02 | -0.23 | | -0.25 | NT_007995 | *BAG4* |
| -1.09 | | -0.02 | 0.08 | | 0.45 | NT_024524 | *NEK3* |
| -1.10 | | -0.18 | 0.40 | | 0.01 | NM_031206 | *LAS1L* |
| -1.12 | | -0.40 | -0.39 | | -0.28 | NT_010966 | *SETBP1* |
| -1.13 | | -0.32 | -0.03 | | 0.04 | NT_037887 | *SSB3* |

| Group III  Genes that only vary with RNAi of FUS and not with RNAi of A1/H/p68 | | | | | | | | |
| --- | --- | --- | --- | --- | --- | --- | --- | --- |
| A1 | FUS | | H | | P68 | | Accession Number | Gene  Name |
| *Log_2_* | | | | | | |
| 0.77 | | 1.67 | | -0.84 | | 0.70 | NT_026437 | *FBXO33* |
| -0.15 | | -1.33 | | -0.11 | | 0.47 | NT_033968 | *EGFR* |
| -0.50 | | -1.40 | | 0.46 | | -0.31 | NT_004487 | *FMOD* |
| 0.20 | | -1.48 | | 0.76 | | 0.38 | NW_922784 | *ADAMTS19* |
| -0.15 | | -1.50 | | -0.40 | | 0.02 | NT_011519 | *GP1BB* |
| -0.31 | | -1.55 | | -0.51 | | 0.52 | NW_927217 | *PSG4* |
| 0.13 | | -1.57 | | -0.69 | | 0.36 | NT_005403 | *SATB2* |
| 0.00 | | -1.78 | | 0.19 | | -0.56 | NW_927317 | *FKBP1A* |
| 0.44 | | -2.03 | | -0.08 | | -0.65 | NT_029289 | *CENTD3* |

| Group IV  Genes that only vary with RNAi of H and not with RNAi of A1/FUS/p68 | | | | | | | | |
| --- | --- | --- | --- | --- | --- | --- | --- | --- |
| A1 | FUS | | H | | P68 | | Accession Number | Gene  Name |
| *Log_2_* | | | | | | |
| 0.30 | | 0.30 | | 7.25 | | -1.91 | NT_004487 | *S100A3* |
| -0.11 | | -0.54 | | 5.76 | | -0.33 | NT_030059 | *ATE1* |
| 0.43 | | 0.23 | | 3.53 | | 0.19 | NT_023935 | *ZFAND5* |
| 0.28 | | -0.07 | | -2.60 | | 0.29 | NW_921618 | *PIP5K3* |
| 0.12 | | -0.27 | | -2.70 | | -0.88 | NT_029419 | *ACVRL1* |
| 0.07 | | -0.18 | | -2.73 | | -0.51 | NT_007819 | *STK17A* |
| -0.11 | | -0.20 | | -2.74 | | 0.24 | NT_016297 | *KLB* |
| 0.06 | | -0.16 | | -2.85 | | -0.05 | NT_011515 | *PRMT2* |
| 1.05 | | 0.69 | | -2.89 | | 0.05 | NT_008583 | *PLEKHK1* |
| -0.53 | | -0.36 | | -3.00 | | 0.46 | NT_021877 | *ESRRG* |
| -0.40 | | -0.42 | | -3.14 | | -0.32 | NT_033903 | *MARK2* |
| -0.58 | | -0.79 | | -3.35 | | -0.17 | NT_025028 | *TCF4* |
| 0.43 | | 0.12 | | -3.64 | | 0.53 | NT_008413 | *TYRP1* |
| 0.10 | | 0.01 | | -3.64 | | 0.27 | NT_008470 | *COL15A1* |
| 0.25 | | 0.11 | | -4.62 | | -0.43 | NT_011520 | *MGAT3* |
| -0.43 | | 0.27 | | -4.71 | | -0.09 | NT_007592 | *ZNF76* |
| -0.48 | | -0.51 | | -5.18 | | -0.57 | NT_079573 | *ARAF* |
| 0.09 | | -0.45 | | -6.53 | | 1.02 | NT_011362 | *CABLES2* |
| -0.55 | | -0.33 | | -7.46 | | -0.96 | NT_022517 | *PCBP4* |

| Group V  Genes that Only vary with RNAi of p68 and not with RNAi of A1/FUS/H | | | | | | | | |
| --- | --- | --- | --- | --- | --- | --- | --- | --- |
| A1 | FUS | | H | | P68 | | Accession Number | Gene  Name |
| *Log_2_* | | | | | | |
| 0.00 | | 0.27 | | -0.37 | | 4.02 | NT_007741 | *PTPRN2* |
| -0.45 | | 0.09 | | 0.73 | | 3.37 | NT_005403 | *B3GALT1* |
| -0.38 | | -1.00 | | -0.19 | | 3.20 | NT_026437 | *C14orf92* |
| -0.14 | | 0.33 | | 0.09 | | 2.84 | NT_011520 | *SEPT3* |
| -0.03 | | 0.12 | | -0.29 | | 2.78 | NT_007933 | *HBP1* |
| -0.29 | | 0.59 | | -0.04 | | 2.70 | NT_033899 | *KIAA0999* |
| 0.39 | | -0.04 | | 0.30 | | -2.91 | NT_011520 | *TOB2* |

*Log_2_* is the log_2_ value of the fold change measuring the effect of the RNAi on the ESTs expresión as compared with the negative control performed with the empty pSuper vector. *Log_2_* higher than 1 were taken as significant values.
